# Puberty disorders among ART-conceived singletons: a Nordic register study from the CoNARTaS group

**DOI:** 10.1093/humrep/deac192

**Published:** 2022-08-26

**Authors:** R Klemetti, B Perry, A K Aaris Henningsen, A Lærke Spangmose, A Pinborg, S Opdahl, L Bente Romundstad, C Bergh, U B Wennerholm, A Tiitinen, M Gissler

**Affiliations:** Department of Public Health and Welfare, Finnish Institute for Health and Welfare (THL), Helsinki, Finland; University of Tampere, Tampere, Finland; The Fertility Clinic, Copenhagen University Hospital, Rigshospitalet, Copenhagen, Denmark; The Fertility Clinic, Copenhagen University Hospital, Rigshospitalet, Copenhagen, Denmark; The Fertility Clinic, Copenhagen University Hospital, Rigshospitalet, Copenhagen, Denmark; Department of Public Health and Nursing, The Norwegian University of Science and Technology, Trondheim, Norway; Department of Public Health and Nursing, The Norwegian University of Science and Technology, Trondheim, Norway; Department of Obstetrics and Gynecology, Institute of Clinical Sciences, Sahlgrenska Academy, University of Gothenburg, Gothenburg, Sweden; Department of Women’s Health, Sahlgrenska University Hospital, Gothenburg, Sweden; Department of Obstetrics and Gynecology, Institute of Clinical Sciences, Sahlgrenska Academy, University of Gothenburg, Gothenburg, Sweden; Department of Women’s Health, Sahlgrenska University Hospital, Gothenburg, Sweden; Department of Obstetrics and Gynecology, Helsinki University Hospital, University of Helsinki, Helsinki, Finland; Department of Molecular Medicine and Surgery, Karolinska Institute, Stockholm, Sweden; INVEST Joint Research Flagship Centre, University of Turku, Turku, Finland; Department of Knowledge Brokers, Finnish Institute for Health and Welfare (THL), Helsinki, Finland

**Keywords:** ART, IVF, ICSI, puberty, puberty disorders, late puberty, early puberty, register-based study

## Abstract

**STUDY QUESTION:**

Do ART-conceived children have an increased risk for puberty disorders?

**SUMMARY ANSWER:**

Both ART-conceived boys and girls had a higher risk of puberty disorders; early puberty was more common among girls and late puberty among boys.

**WHAT IS KNOWN ALREADY:**

Some physiological differences in growth and metabolism have been reported for ART-conceived children compared to non-ART-conceived children. Knowledge on pubertal development and disorders in ART-conceived children is limited.

**STUDY DESIGN, SIZE, DURATION:**

A register-based cohort study was carried out including data from 1985 to 2015. The Committee of Nordic Assisted Reproductive Technology and Safety (CoNARTaS) study population consists of all live and stillborn children, as well as their mothers, registered in the Medical Birth Registers during the study period in Denmark, Sweden, Finland and Norway.

**PARTICIPANTS/MATERIALS, SETTING, METHODS:**

A total of 122 321 ART-conceived singletons and 6 576 410 non-ART singletons born in Denmark (1994–2014), Finland (1990–2014), Norway (2002–2015) and Sweden (1985–2015) were included. Puberty disorders were defined using International Classification of Diseases and Related Health Problems (ICD)-9/ICD-10 codes and classified in the following groups: late puberty (6268/E30.0), early puberty (2591 and 2958/E30.1 and E30.8) and unspecified disorders (V212 and V579/E30.9 and Z00.3 as well as Z51.80 for Finland). The results in Cox regression were adjusted for maternal age, parity, smoking, gestational diabetes, chronic hypertension, hypertensive disorders during pregnancy and country, and further for either gestational age, birthweight, small for gestational age or large for gestational age.

**MAIN RESULTS AND THE ROLE OF CHANCE:**

There were 37 869 children with diagnoses related to puberty disorders, and 603 of them were born after ART. ART-conceived children had higher risks for early (adjusted hazard ratio (aHR) 1.45, 95% CI: 1.29–1.64) and late puberty (aHR 1.47, 95% CI: 1.21–1.77). Girls had more diagnoses related to early puberty (aHR 1.46, 95% CI: 1.29–1.66) and boys with late puberty (aHR 1.55, 95% CI: 1.24–1.95).

**LIMITATIONS, REASONS FOR CAUTION:**

Using reported puberty disorders with ICD codes in health care registers might vary, which may affect the numbers of cases found in the registers. Register data may give an underestimation both among ART and non-ART-conceived children, especially among non-ART children, who may not be as carefully followed as ART-conceived children. Adjustment for causes and duration of infertility, mothers’ own puberty characteristics and BMI, as well as children’s BMI, was not possible because data were not available or data were missing for the early years. It was also not possible to compare ART to non-ART siblings or to study the pubertal disorders by cause of subfertility owing to a small number of discordant sibling pairs and a large proportion of missing data on cause of subfertility.

**WIDER IMPLICATIONS OF THE FINDINGS:**

This large, register-based study suggests that ART-conceived children have a higher risk for puberty disorders. However, the mechanisms of infertility and pubertal onset are complex, and ART is a rapidly advancing field with various treatment options. Studying the pubertal disorders of ART-conceived offspring is a continuing challenge.

**STUDY FUNDING/COMPETING INTEREST(S):**

This work was supported by the Nordic Trial Alliance: a pilot project jointly funded by the Nordic Council of Ministers and NordForsk (71450), the Central Norway Regional Health Authorities (46045000), the Nordic Federation of Obstetrics and Gynaecology (NF13041, NF15058, NF16026 and NF17043), the Interreg Öresund-Kattegat-Skagerrak European Regional Development Fund (ReproUnion project), the Research Council of Norway’s Centre of Excellence funding scheme (262700), the Swedish state under the agreement between the Swedish government and the county councils, the ALF-agreement (ALFGBG-70940) and FLUX Consortium ‘Family Formation in Flux—Causes, Consequences and Possible Futures’, funded by the Strategic Research Council, Academy of Finland (DEMOGRAPHY 345130). The funders had no role in study design, data collection and analysis, decision to publish or preparation of the manuscript. The authors have no conflicts of interest to disclose.

**TRIAL REGISTRATION NUMBER:**

N/A.

## Introduction

The safety of ART and their potential effects on long-term health are areas of growing interest. It is known that ART-conceived babies have a higher risk of preterm birth, low birthweight, being small and large for gestational age (SGA, LGA), and perinatal death compared with non-ART babies (Klemetti  *et al*., 2006; Pinborg *et al.*, 2013; Qin *et al.*, 2017; Westvik-Johari *et al.*, 2021). Low birthweight and SGA might predispose offspring to adiposity or later obesity, and thus increased risk of earlier onset of puberty. Rapid postnatal growth may be more common in ART-conceived children and, regardless of cause, seems to be correlated with early onset of puberty (Ceelen *et al.*, 2009; Hvidt *et al.*, 2019). According to the developmental origins of health and disease hypothesis, these adverse birth outcomes may have long-term consequences for adult health (Barker *et al.*, 2002). Many children conceived by ART have now reached adulthood, providing the opportunity to study these potential consequences.

Research evaluating outcomes in adolescents conceived with ART shows few adverse outcomes (Hart and Norman, 2013). Some physiological differences in growth and metabolism (Catford *et al.*, 2018) and a higher risk for neurodevelopmental problems (Bergh and Wennerholm, 2020; Rissanen *et al.*, 2020) among ART‐conceived adolescents and young adults have been reported. However, most studies on neurocognitive development or autism spectrum disorder among ART-conceived offspring do not show higher risks after adjusting for multiple births (Bergh and Wennerholm, 2020; Rönö *et al.*, 2022).

In the general population, trends suggest that girls are entering puberty earlier, and that boys may be entering puberty earlier, as well (Euling *et al.*, 2008; Sørensen *et al.*, 2010; Papadimitriou, 2016). One hypothesized cause of changes in pubertal timing is the increasing prevalence of childhood obesity owing to a relative overnutrition (Solorzano and McCartney, 2010; Toppari and Juul 2010; Biro and Kiess, 2016). It has been discussed that childhood obesity could lead to late puberty among boys and early signs of puberty (thelarche) among girls. The early signs of puberty among girls might be linked to central activation of the GnRH-gonadotrophin axis, increased peripheral aromatization of adrenal androgens or hyperinsulinemia (Solorzano and McCartney, 2010). However, the obesity epidemic was unable to explain the concerning trends (Toppari and Juul 2010). Exposure to environmental chemicals has been considered to have an important role by having direct hormone actions or/and interfering with hormone synthesis, action and metabolism within the body (Solorzano and McCartney, 2010; Toppari and Juul 2010; Biro and Kiess 2016). It has also been discussed whether the earlier onset of puberty among girls might be partly linked to maternal obesity, maternal smoking (Syme *et al.*, 2010), gestational diabetes (Kawasaki *et al.*, 2018) or hypertensive disorders of pregnancy (Ogland *et al.*, 2011; Lunddorf *et al.*, 2020).

Early puberty in both boys and girls may be associated with a higher risk for immediate and long-term health consequences including dysmetabolism, psychosocial issues and chronic disease (Aris *et al.*, 2022). Delayed puberty may also have both immediate and long-term health effects on both girls and boys, but these effects have not been as clearly established (Zhu and Chan, 2017). Overall, puberty disorders may signal an underlying health condition and it is important to identify which disorders require appropriate hormone therapies, and which are partial or slow, progressive forms that need monitoring but no treatment (Farello *et al.*, 2019).

Knowledge on pubertal development and disorders in ART-conceived children is limited. Bone age appeared to be advanced in pubertal IVF-conceived girls compared to non-ART controls. However, the pubertal stage and age at menarche were similar (Ceelen *et al.* 2008). Pubertal girls born after ICSI have been more prone to central, peripheral and total adiposity and boys to peripheral adiposity compared with non-ART girls and boys (Belva et al. 2012b). In a nationwide cohort study, neither type of assisted reproduction nor time to pregnancy had clinically important associations with the overall timing of puberty in boys and girls (Ernst *et al.*, 2019).

Previous studies have been small, either examining adolescents clinically or by using web-based questionnaires. This study aims to investigate the risk of puberty disorders among ART-conceived children, both girls and boys, as available in existing health care registers, using data from four Nordic countries. Known risks for pubertal disorders, such as low birthweight and SGA, are more common in ART-conceived children, thus we hypothesize that ART-conceived children may have a higher risk for puberty disorders.

## Materials and methods

### Study population and participants

The Committee of Nordic Assisted Reproductive Technology and Safety (CoNARTaS) study population consists of all live and stillborn children, as well as their mothers, registered in the Medical Birth Registers during the study period in Denmark, Sweden, Finland and Norway (Opdahl *et al.*, 2020). The information on ART conception was taken from national ART registers (Denmark 1994–2014, Sweden 1985–2015) and Medical Birth Registers (Finland 1990–2014 and Norway 1984–2015). In total, the CoNARTaS cohort contains information on 172 161 ART-conceived and 7 681 797 non-ART children.

To enable long-term follow-up, data on all children and mothers were linked to data from other health registers providing information on the use of specialized health services, diagnoses and causes of death. Data were linked at an individual level using the national identity number assigned to all residents in each country at birth or immigration (Opdahl *et al.*, 2020).

Socio-demographic data on education, death and immigration were retrieved from Central Population Registers and Statistical Offices. Information on puberty disorders was taken from the national patient registers. Hospital admissions have been registered at an individual level since 1977 in Denmark, 1967 in Finland, 2008 in Norway and 1987 in Sweden. Outpatient visits in public hospitals have been included since 1995 in Denmark, 1998 in Finland and 2008 in Norway. Data for Sweden include all outpatient specialized care visits since 2001. For each contact, at least one International Statistical Classification for Diseases and Related Health Problems (ICD) diagnosis is registered.

Early puberty is defined as onset of the first sign of puberty and/or early appearance of secondary sexual characteristics before the age of 8 years in girls and 9 years in boys, or as menarche before the age of 10 years in girls (Alotaibi, 2019). Delayed puberty is defined as the lack of signs of secondary sexual characteristics by the age of 13 years in girls (lack of thelarche) and 14 years in boys (testicular volume < 3 ml). In our study, puberty disorders were defined using ICD-9/ICD-10 codes and classified in the following groups: late puberty (6268/E30.0), early puberty (2591 and 2958/E30.1 and E30.8) and unspecified diagnoses related to puberty (disorders) (V212 and/E30.9 and Z00.3 as well as V579/Z51.80 for Finland). Diagnoses were given in a specialist care setting (either inpatient or outpatient) after the child had been referred to specialized care from a primary care setting. For all visits, a primary ICD code was reported at a minimum.

Since Norway started including identification numbers in their patient register in 2008, Norwegian children born in 2001 or earlier were excluded. The final study data contained information on 122 321 ART-conceived live born singletons and 6 574 731 non-ART live born singletons in Denmark (1994–2014), Finland (1990–2014), Norway (2002–2015) and Sweden (1985–2015).

### Statistical analyses

We used Cox regression adjusted for potential confounders to estimate hazard ratios (HRs) with 95% CI for puberty disorders. In the first model, we used country, maternal age, parity, maternal pregestational diabetes (type 1 and 2 diabetes) and maternal smoking during pregnancy as confounding factors (Model 1). Maternal smoking was considered to be a confounder through biological influence and was additionally used as a proxy for confounding from maternal socioeconomic position, since data on maternal educational level at childbirth were not available for all countries. We further added adjustment for potential mediators, such as maternal chronic hypertension and hypertensive disorders of pregnancy (Model 2), to account for the potential of compromised fetal blood flow leading to intrauterine growth restriction (IUGR) or SGA, as well as adding in the model either gestational age (Model 3), birthweight (Model 4) or SGA (<10th percentile) and LGA (>90th percentile) (Model 5). For variables other than maternal smoking, where we included a separate category of cases with no information, the number of missing cases was small and complete case analyses without imputations were made. Follow-up started at birth and ended at first diagnosis, death, emigration (not available for Finland) or the age of 20 years. Analyses for IVF versus ICSI and fresh versus frozen embryo transfer were done for Denmark, Norway and Sweden, excluding Finland, since these data were not available for Finland. All analyses were made separately for girls and boys. We performed analyses for known or unknown cause of infertility with data from Denmark, Norway and Sweden. Data were too sparse to be able to estimate the associations according to the cause of infertility by sex and type of ART. We also aimed to perform a sibling analysis, but our data were too small for analysis, as there were only 8944 sibling-boy-pairs and 7860 sibling-girl-pairs with discordant exposure to ART (data not shown).

### Ethical and legal approvals

Approvals for data retrieval and linkage were obtained in each country. In Denmark and Finland, ethical approval is not required for scientific projects solely based on registry data. In Norway, ethical approval was given by the Regional Committee for Medical and Health Research Ethics (REK-Nord, 2010/1909). In Sweden, approval was obtained from the Ethical committee in Gothenburg, Dnro 214-12, T422-12, T516-15, T233-16, T300-17, T1144-17, T1071-18, T121-18 and T2019 02347 (2019-04-23). All register-keeping organizations gave their permission to use their data in this study.

## Results

### Characteristics of the populations

The mothers of ART-conceived children were older, more often primiparas and non-smokers than mothers of non-ART children (Table I). The mean follow-up time was 8.5 (SD 5.7) years for ART-conceived children and 12.2 (SD 6.6) years for non-ART children.

**Table I:** Background characteristics of mothers and their ART and non-ART children born in 1985–2015 in Denmark, Finland, Norway and Sweden.

	ART (n = 122 321)		Non-ART (n = 6 574 410)		Total (n = 6 696 731)	
	N	%	N	%	N	%
**Sex**						
Boy	62 531	51.1	3 374 701	51.3	3 437 232	51.3
Girl	59 788	48.9	3 199 645	48.7	3 259 433	48.7
Unknown	2	0	64	0	66	0
Total	122 321	100	6 574 410	100	6 696 731	100
**Country**						
Denmark	31 061	25.4	1 268 842	19.3	1 299 903	19.4
Finland	22 038	18	1 431 427	21.8	1 453 465	21.7
Norway	19 151	15.7	785 417	11.9	804 568	12
Sweden	50 071	40.9	3 088 724	47	3 138 795	46.9
Total	122 321	100	6 574 410	100	6 696 731	100
**Maternal age at childbirth (years)**						
Below 25	1630	1.3	1 021 645	15.5	1 023 275	15.3
25–29	17 332	14.2	2 130 993	32.4	2 148 325	32.1
30–34	47 376	38.7	2 175 731	33	2 223 107	33.2
35–39	43 325	35.4	1 020 063	15.5	1 063 388	15.9
40 or more	12 658	10.3	225 970	3.4	238 628	3.6
Unknown	0	0	8	0	8	0
Total	122 321	100	6 574 410	100	6 696 731	100
**Maternal parity**						
Primipara	82 840	67.7	2 754 131	42	2 836 971	42.4
Multipara	38 829	31.7	3 784 717	57.6	3 823 546	57.1
Unknown	652	0.5	35 562	0.5	36 214	0.5
Total	122 321	100	6 574 410	100	6 696 731	100
**Maternal smoking**						
No	108 195	88.5	5 188 156	78.9	5 296 351	79.1
Yes	6736	5.5	927 436	14.1	934 172	13.9
NA^*^	7390	6	458 818	7	466 208	7
Total	122 321	100	6 574 410	100	6 696 731	100
**Any pregestational diabetes**						
No	122 001	99.7	6 559 290	99.8	6 681 291	99.8
Yes	320	0.3	15 120	0.2	15 440	0.2
Total	122 321	100	6 574 410	100	6 696 731	100
**Hypertensive disorders of pregnancy**						
No	114 052	93.2	6 304 770	95.9	6 418 822	95.9
Yes	8269	6.8	269 640	4.1	277 909	4.1
Total	122 321	100	6 574 410	100	6 696 731	100
**Chronic hypertension**						
No	120 877	98.8	6 528 006	99.3	6 648 883	99.3
Yes	1444	1.2	46 404	0.7	47 848	0.7
Total	122 321	100	6 574 410	100	6 696 731	100
**BMI kg/m^2^**						
Below 20	8359	6.8	485 853	7.4	494 212	7.4
20–24	42 753	35	1 952 619	29.7	1 995 372	29.8
25–29	18 823	15.4	798 407	12.1	817 230	12.2
30 or more	7543	6.2	372 043	5.7	379 586	5.7
Unknown	44 843	36.7	2 965 488	45.1	3 010 331	45
Total	122 321	100	6 574 410	100	6 696 731	100
**SGA** ^**^						
No	107 191	87.6	5 878 288	89.4	5 985 479	89.4
Yes	14 638	12	644 025	9.8	658 663	9.8
Unknown	492	0.4	52 097	0.8	52 589	0.8
Total	122 321	100	6 574 410	100	6 696 731	100
**LGA** ^***^						
No	111 242	90.9	5 881 224	89.5	5 992 466	89.5
Yes	10 587	8.7	641 089	9.8	651 676	9.7
Unknown	492	0.4	52 097	0.8	52 589	0.8
Total	122 321	100	6 574 410	100	6 696 731	100
**Preterm <37 gestational weeks**						
No	112 314	91.8	6 213 870	94.5	6 326 184	94.5
Yes	9726	8	318 247	4.8	327 973	4.9
Unknown	281	0.2	42 293	0.6	42 574	0.6
Total	122 321	100	6 574 410	100	6 696 731	100

* NA = unknown or no permission to give information (Norway).

** SGA = small for gestational age, <10th percentile.

*** LGA = large for gestational age, >90th percentile.

### Puberty disorders

There were 37 869 children with diagnoses related to puberty disorders, of which 603 were ART-conceived children. The crude incidences of late puberty (ART 0.9/1000, non-ART 1.9/1000), and unspecified puberty disorders (ART 1.6/1000, non-ART 2.0/1000) per 1000 livebirths were lower among ART-conceived children, but the incidence of early puberty was higher among ART children (ART 2.4/1000, non-ART 2.1/1000) (Table II). Early puberty and unspecified puberty disorders were more common among girls than boys—both among ART-conceived and non-ART children. In contrast, late puberty was more common among boys, regardless of conception method.

**Table II:** The number and incidence (per 1000) of puberty disorders among ART and non-ART children born in 1985–2015 in Denmark, Finland, Norway and Sweden by sex.*

	ART (n = 122 321)			Non-ART (n = 6 574 410)		
	Boys	Girls	Total	Boys	Girls	Total
**Puberty disorder**	(n = 62 531)	(n = 59 788)	(n = 122 319)	(n = 3 374 701)	(n = 3 199 645)	(n = 6 574 346)
Late	79	35	114	8972	3213	12 185
Early	26	272	298	1591	11 914	13 505
Unspecified	65	131	196	5445	7565	13 010
**Per 1000**						
Late	1.3	0.6	0.9	2.7	1.0	1.9
Early	0.4	4.6	2.4	0.5	3.8	2.1
Unspecified	1.0	2.2	1.6	1.6	2.4	2.0

* Excluding two ART children and 64 non-ART children with unknown sex.

After adjusting for maternal age, parity, smoking, pregestational diabetes and country in Model 1, ART-conceived children had a higher risk for all studied puberty disorders compared to non-ART children (Table III). Further adjustment for maternal chronic hypertension and hypertensive disorders of pregnancy (Model 2) as well as gestational age (Model 3), birthweight (Model 4) and SGA or LGA (Model 5) of children attenuated the results, but the associations remained statistically significant.

**Table III:** The crude and adjusted hazard ratios with 95% CI for puberty disorders among ART children (n = 122 321) compared to non-ART children (n = 6 574 410) born in 1985–2019 in Denmark, Finland, Norway and Sweden, by sex.

		Crude		**Model 1** ^*^		**Model 2** ^**^		**Model 3** ^***^		**Model 4** ^****^		**Model 5** ^*****^	
	Puberty disorder	HR	95% CI	HR	95% CI	HR	95% CI	HR	95% CI	HR	95% CI	HR	95% CI
**All**	Late	1.64	1.36–1.97	1.47	1.21–1.77	1.47	1.21–1.87	1.46	1.21–1.77	1.46	1.21–1.77	1.47	1.21–1.78
	Early	1.86	1.66–2.08	1.45	1.29–1.64	1.45	1.29–1.63	1.43	1.27–1.60	1.39	1.23–1.56	1.44	1.28–1.62
	Unspecified	1.42	1.23–1.63	1.34	1.15–1.55	1.33	1.15–1.54	1.30	1.13–1.512	1.27	1.10–1.47	1.32	1.14–1.53
**Boys**	Late	1.68	1.34–2.09	1.55	1.24–1.95	1.55	1.24–1.95	1.55	1.23–1.95	1.53	1.22–1.93	1.55	1.23–1.95
	Early	1.73	1.19–2.51	1.37	0.93–2.03	1.36	0.92–2.02	1.30	0.88–1.93	1.26	0.84–1.88	1.35	0.91–2.01
	Unspecified	1.26	0.99–1.60	1.30	1.02–1.66	1.34	1.02–1.70	1.26	0.99–1.61	1.25	0.98–1.60	1.29	1.01–1.64
**Girls**	Late	1.58	1.13–2.20	1.31	0.93–1.86	1.32	0.93–1.87	1.29	0.91–1.83	1.31	0.93–1.85	1.32	0.93–1.87
	Early	1.87	1.67–2.11	1.46	1.29–1.66	1.46	1.29–1.65	1.44	1.28–1.63	1.43	1.26–1.61	1.45	1.28–1.64
	Unspecified	1.51	1.27–1.80	1.35	1.13–1.62	1.35	1.12–1.62	1.32	1.10–1.59	1.29	1.07–1.55	1.34	1.12–1.61

* Model 1 adjusted for maternal age, parity, smoking, pregestational diabetes and country.

** Model 2 adjusted for maternal age, parity, smoking, pregestational diabetes, chronic hypertension, hypertensive disorders of pregnancy and country.

*** Model 3 adjusted for maternal age, parity, smoking, pregestational diabetes, chronic hypertension, hypertensive disorders of pregnancy, gestational age and country.

**** Model 4 adjusted for maternal age, parity, smoking, pregestational diabetes, chronic hypertension, hypertensive disorders of pregnancy, birth weight and country.

***** Model 5 adjusted for maternal age, parity, smoking, pregestational diabetes, chronic hypertension, hypertensive disorders of pregnancy, small/large for gestational age and country.

HR, hazard ratio.

In sex-specific analyses, the crude risks for early, late and unspecified puberty disorders were higher among ART-conceived girls than non-ART girls, as were the crude risks for early and late puberty among ART-conceived boys compared to non-ART boys (Table III). For boys, the risk for early puberty was not significantly higher after background adjustment (Model 1) and further adjustment for the mediators (Models 2–5), and the association with unspecified puberty disorders was not statistically significant after adjusting for gestational age (Model 3) and birthweight (Model 4). For girls, all risks remained significantly higher in different models, except for late puberty.

### Puberty disorders according to different ART methods

The incidences of puberty disorders did not significantly differ between children born after IVF and ICSI (Table IV). However, compared to non-ART children, after adjusting for background characteristics, ICSI was associated with higher risk for late puberty (adjusted HR (aHR) 3.64, 95% CI: 2.58–5.13) than IVF (aHR 1.37, 95% CI: 1.02–1.85). By sex, ICSI was associated with statistically significant increased risks for boys and girls for all studied puberty disorders except unspecified puberty among boys; IVF was associated with increased risk for early puberty among girls and late puberty among boys. No differences were seen when analyzing incidence of puberty disorders by known versus unknown causes of infertility (data not shown).

**Table IV:** The incidence and hazard ratios with 95% CI for puberty disorders among IVF (n = 56 176) and ICSI (n = 38 531) children compared to non-ART (n = 5 142 983) children born in 1985-2015 in Denmark, Norway and Sweden.

			Crude		**Adjusted** ^*^					
Puberty disorder	Type of ART	Ratio 1/1000	All		All		Boys		Girls	
			HR	95% CI	HR	95% CI	HR	95% CI	HR	95% CI
Late	IVF	0.8	1.53	1.15–2.04	1.37	1.02–1.85	1.53	1.09–1.69	1.00	0.54–1.87
	ICSI	0.9	3.83	2.73–5.37	3.64	2.58–5.13	4.63	3.04–7.05	2.49	1.37–4.51
	Non-ART	1.8	1		1		1		1	
Early	IVF	2.7	1.68	1.43–1.97	1.36	1.15–1.61	1.28	0.72–2.28	1.40	1.17–1.67
	ICSI	2.6	1.95	1.60–2.38	1.62	1.32–1.99	1.98	1.03–3.84	1.52	1.22–1.89
	Non-ART	2.2	1		1		1		1	
Unspecified	IVF	1.8	1.33	1.09–1.62	1.25	1.02–1.54	1.22	0.87–1.73	1.29	0.99–1.67
	ICSI	2.2	1.82	1.43–2.31	1.63	1.27–2.09	1.49	0.95–2.34	1.65	1.23–2.23
	Non-ART	2.2	1		1		1		1	

* Adjusted for country, maternal age, smoking, parity and pregestational diabetes.

HR, hazard ratio.

The incidence of puberty disorders among ART-conceived children was higher after fresh than after frozen embryo transfers (Table V). However, compared to non-ART children, the aHR for fresh and frozen embryo transfers did not differ significantly from each other.

**Table V:** The incidence (1/1000) and hazard ratios with 95% CI for puberty disorders among fresh (n = 80 896) and frozen (n = 18 711) embryo transfers comparing ART children to non-ART children (n = 5 142 983) born in 1985–2015 in Denmark, Norway and Sweden.

Puberty disorder	Type of ART	Ratio 1/1000	Crude		**Adjusted** ^*^	
			HR	95% CI	HR	95% CI
Late	Fresh ART	1.1	2.12	1.78–2.74	1.98	1.58–2.48
	Frozen ART	0.1	0.58	0.15–2.33	0.57	0.14–2.28
	Non-ART	1.8	1		1	
Early	Fresh ART	2.9	1.80	1.58–2.05	1.42	1.24–1.62
	Frozen ART	1.7	1.62	1.14–2.30	1.49	1.04–2.14
	Non-ART	2.2	1		1	
Unspecified	Fresh ART	1.8	1.38	1.17–1.63	1.35	1.13–1.60
	Frozen ART	1.3	1.88	1.27–2.78	1.56	1.04–2.35
	Non-ART	2.2	1		1	

* Adjusted for country, maternal age, smoking, parity and pregestational diabetes.

HR, hazard ratio.

## Discussion

In this large register-based study, ART-conceived singletons had a higher risk of puberty disorders compared with non-ART singletons. These risks were higher among both ART-conceived girls and boys. Maternal characteristics and perinatal outcomes partly attenuated the associations. Compared to non-ART girls and non-ART boys, ART-conceived girls had higher risk for early puberty and ART-conceived boys had a higher risk for late puberty, and risks after ICSI were higher than after IVF.

The strengths of this study are that it is based on four large Nordic national datasets with high coverage and validity, and that it uses population-based registries, which reduce the risk of selection bias (Laugesen *et al.*, 2021). Even though puberty disorders are not common, our results are based on 603 ART-conceived children with puberty disorders. To our knowledge, this is the largest reported study on the issue. While there may be variations in how professionals use and report ICD codes of puberty disorders, this variation would be expected to result in non-differential misclassification and bias toward the null. Nationwide studies on pubertal disorders using register data are sparse. A Danish study on the incidence of precocious puberty used ICD-10 codes, but the authors noted that none of the ICD-10 codes were validated by investigation of hospital records (Teilmann *et al.*, 2005), which may have affected the number of cases included (Bräuner *et al.*, 2020). In our study, some of the puberty disorders were coded as unspecified, and we could not determine whether these were cases in which the final diagnosis was unknown or children for whom no diagnosis could be confirmed.

It has been suggested that poorer health outcomes or higher use of health services by ART-conceived children might partly be explained by parental worries or higher socioeconomic position (Klemetti *et al.*, 2006). It is possible that puberty disorders may be diagnosed more often among ART-conceived children for a similar reason. Therefore, the risks for non-ART children may be an underestimation. We were not able to adjust for parental socioeconomic position, but we used adjustment for maternal smoking as a proxy (Grøtvedt *et al.*, 2017; de Wolff *et al.*, 2019; Rumrich *et al.*, 2019). Although comparing ART-conceived children to their non-ART-conceived siblings may reduce bias from parental worries and socioeconomic status, our data were too small for sibling analyses.

While we adjusted for several known confounders, such as pregestational diabetes and chronic hypertension, we could not adjust for cause of infertility, duration of infertility, the mothers’ own puberty characteristics and BMI or children’s own BMI, because data were not available or data were missing for the early years. Other limitations of our study include missing information on diagnoses given in primary health care only and on paternal characteristics and a relatively short follow-up time, especially among ART-conceived children. The difference in follow-up time between the ART and non-ART groups has been considered in the aHR.

In our study, incidences of late puberty were higher among boys and early puberty among girls, both among ART and non-ART children. The onset and progression of puberty are determined by genetic and epigenetic factors and are influenced by extrinsic factors such as environmental, chemical, nutritional and cultural influences (Kiess *et al.*, 2016). During recent decades, earlier onset of puberty among girls in the general population has been reported (Aksglaede *et al.*, 2009; Eckert-Lind *et al.*, 2020). Among boys, the trends are less clear. Some studies suggest an increasing incidence of late puberty among boys (Euling *et al.*, 2008; Toppari and Juul, 2010), but results from the latest Scandinavian studies are contradictory (Sørensen *et al.*, 2010; Ohlsson *et al.*, 2019). These trends are partly believed to be related to increased childhood obesity (Solorzano and McCartney, 2010). Fetal exposure to tobacco smoke might advance the onset of puberty in girls and boys, as maternal smoking is associated with pubertal milestones in both girls and boys (Brix *et al.*, 2019).

Our results show that ART-conceived girls have a higher proportion of diagnoses related to early puberty than non-ART-conceived girls. This is in contradiction to previous studies in which mean age of menarche and pubarche in girls were similar regardless of conception method (Ceelen *et al.*, 2008; Belva et al., 2012a). Early reports on pubertal development of IVF-conceived children used retrospective self-scoring questionnaires and did not show any pubertal abnormalities (Beydoun *et al.*, 2011). However, some minor differences in pubertal development among ART-conceived children have been reported. In a Dutch clinical study, bone age appeared to be advanced in pubertal IVF-conceived girls compared with non-ART controls (Ceelen *et al.*, 2008). On the contrary, breast development was less advanced in ICSI-conceived females than non-ART peers, even after adjusting for known confounders (Belva et al., 2012b). It is possible that these discrepancies are due to the smaller size of previous studies, as well as our data being sourced from routinely collected population-based national health care registers.

Compared to non-ART children, ICSI-conceived children in our study had a higher risk for pubertal disorders than IVF-conceived children, especially in ICSI-conceived boys presenting with late puberty. Previously, no signs of delayed puberty in ICSI-conceived children compared to other children were observed (Belva et al., 2012b). In a prospective study including 274 singleton ICSI-conceived adolescents (141 girls, 133 boys) and 273 non-ART controls (142 girls, 131 boys), age-adequate pubertal maturation was reported (Sonntag *et al.*, 2020). However, there was a tendency toward lower inhibin B levels, significantly higher estradiol levels, and a lower testosterone-to-estradiol-ratio in male adolescents. In a Belgian study of semen quality in 54 young men born after ICSI (Belva *et al.*, 2016), median sperm concentration, total sperm count and total motile sperm count were significantly lower than in 57 non-ART peers. In another Belgian cohort study of 71 young women conceived by ICSI, the antral follicle count and circulating reproductive hormone levels were similar to non-ART controls (Belva *et al.*, 2017). However, they were unable to separate the effects of the ICSI procedure and underlying paternal infertility (Belva *et al.*, 2017). In our study, we were also unable to study pubertal disorders by the cause of infertility separated by the sex and type of ART. However, future studies should investigate whether the higher risks for pubertal disorders, especially among ICSI-conceived boys, might be related to transgenerational inheritance of male infertility (Catford *et al.*, 2018). Our results show a higher risk for pubertal disorders among ICSI-conceived girls, as well, suggesting that cause of infertility and inheritance might play a role regardless of the child’s sex and that the ICSI procedure itself might also affect offspring reproductive function.

The higher risks for puberty disorders among ART-conceived children in our study were partly explained by the measured maternal risk factors, as well as poorer perinatal outcomes, such as low birthweight and preterm birth, as found in earlier studies regarding the pubertal disorders among female offspring (Syme *et al.*, 2010; Ogland *et al.*, 2011; Kawasaki *et al.*, 2018; Lunddorf *et al.*, 2020). Children with IUGR, especially if they experience catch-up growth in early life, have a higher risk for long-term problems, including short stature and the development of metabolic syndrome and cardiovascular diseases. Small size at birth and rapid infant growth were associated with early pubertal age, most consistent and pronounced in girls (Hvidt *et al.*, 2019). However, our results remained significant even after adjustments, suggesting that higher risks might be partly related to residual confounding factors such as infertility or the ART treatment itself (Guo *et al.*, 2017). In a Danish nationwide cohort (15 819 children), neither time to pregnancy nor type of ART treatment had clinically relevant implications for mean age at onset of puberty in girls and boys (Ernst *et al.*, 2019). However, the authors found that the mean age of puberty onset was slightly lower in females and slightly higher in males among children born to parents with both untreated and treated subfertility.

## Conclusion

In conclusion, both ART-conceived girls and boys had a higher risk of pubertal disorders compared with their non-ART peers. Measured maternal characteristics, gestational age and birthweight partly attenuated the associations. However, as the mechanisms of infertility and pubertal onset are complex and ART is a rapidly advancing field with various treatment options, studying the pubertal disorders of ART-conceived offspring is a continuing challenge.

## Data Availability

The data underlying this article cannot be shared publicly. The researchers have received permissions from the register keeping organizations to use their sensitive health data in this study. The data cannot be shared without receiving the necessary permissions from the four study countries. The corresponding author can be contacted to get advice on the process.
